# Primary adrenal insufficiency in patients with bilateral adrenal metastases treated with curative ablative radiation therapy: A comprehensive review of literature and expert agreements for radiation practice

**DOI:** 10.1016/j.ctro.2026.101195

**Published:** 2026-05-25

**Authors:** Alexander Bennassi, Kamel Debbi, Nicolas Giraud, Lahcène Belaïdi, Anna Durigova, Emanuela Salati, Sami Frikha, Fatima Zahra Bellefkih, Wassim Ksouri, Nisrine El Hayel, Pelagia Tsoutsou, Fady Geara, Dusanka Tesanovic, Shaïma El Chammah, Mahmut Ozsahin, Céline Droumaguet, Yazid Belkacemi

**Affiliations:** a AP-HP. Department of Radiation Oncology and Henri Mondor Breast Center, Henri Mondor University Hospital, 1 rue Gustave Eiffel, 94000 Créteil, France; b Mondor Institute of Biomedical Research (IMRB), INSERM U955, i-Biot and University of Paris East Créteil (UPEC), France; c Department of Radiation Oncology, Bordeaux University Hospital (CHU), Haut-Lévêque Hospital, Avenue de Magellan, 33604 Pessac, France; d Department of Radiation Oncology, Amsterdam UMC, Amsterdam, Netherlands; e Department of Oncology, Rte du Vieux Séquoia 20, 1847 Rennaz, Switzerland; f Department of Radiation Oncology, Av. de la Roseraie 53, 1205 Genève, Switzerland; g Department of Radiation Oncology, Oncology Institute, Cleveland Clinic Abu Dhabi, Abu Dhabi, United Arab Emirates; h Radiotherapy Department, Institute for Oncology of Vojvodina, Sremska Kamenica, Serbia; i Department of Radiation Oncology, Rte du Vieux Séquoia 20, 1847 Rennaz, Switzerland; j Department of Internal Medicine, Henri Mondor Hospital, 1 rue Gustave Eiffel, 94000 Créteil, France

**Keywords:** Adrenal metastases, Stereotactic body radiotherapy, Primary adrenal insufficiency, Bilateral adrenal irradiation, Radiation-induced toxicity, Endocrine side effects

## Abstract

**Background:**

Bilateral adrenal metastases are rare but clinically significant. While stereotactic body radiotherapy (SBRT) provides excellent local control, the risk of post-treatment primary adrenal insufficiency (PAI) after bilateral irradiation remains poorly defined and lacks standardized management guidelines.

**Methods:**

We performed a comprehensive literature review following PRISMA principles, using PubMed and Embase databases (January 1956 to June 2025). Studies including patients with bilateral adrenal metastases treated with radiotherapy, or unilateral irradiation in the setting of a solitary adrenal gland, were eligible. Data on treatment characteristics, incidence of PAI, diagnostic approaches, and management were extracted. In parallel, a multidisciplinary expert panel developed expert opinion–based practical recommendations for the screening, treatment, and follow-up of PAI.

**Results:**

Thirty studies comprising 156 patients treated with bilateral adrenal irradiation (or irradiation of a solitary adrenal gland) were included. PAI was reported in 47 patients (30%), although systematic endocrine evaluation was performed in only a minority of studies, suggesting potential underestimation. Time to onset ranged from 3 to 30 months. Reporting of diagnostic work-up, management, and follow-up was highly heterogeneous, and treatment details were frequently lacking. No study identified reliable dosimetric predictors of PAI, and no association between radiation dose parameters and adrenal insufficiency was demonstrated.

**Conclusions:**

PAI following bilateral adrenal radiotherapy is likely under-recognized and under-reported, with clinically meaningful incidence and potential life-threatening consequences. In the absence of robust data, systematic hormonal assessment, careful treatment planning, and structured long-term follow-up are essential. We propose practical, expert-based recommendations to guide clinical management, while emphasizing the need for prospective studies to better define incidence, risk factors, and optimal prevention strategies.

## Highlights

• Primary adrenal insufficiency is underrecognized after bilateral adrenal SBRT or RT to a solitary adrenal gland.
• Systematic screening is rarely performed despite non-specific but serious symptoms.
• Patients receiving bilateral adrenal SBRT or RT to a solitary adrenal gland are at high risk of adrenal insufficiency.
• No consensus guidelines exist; we propose an expert-informed follow-up algorithm.
• Clinician and patient education are key to preventing adrenal crises in this setting.

## Graphical abstract

**Figure:**
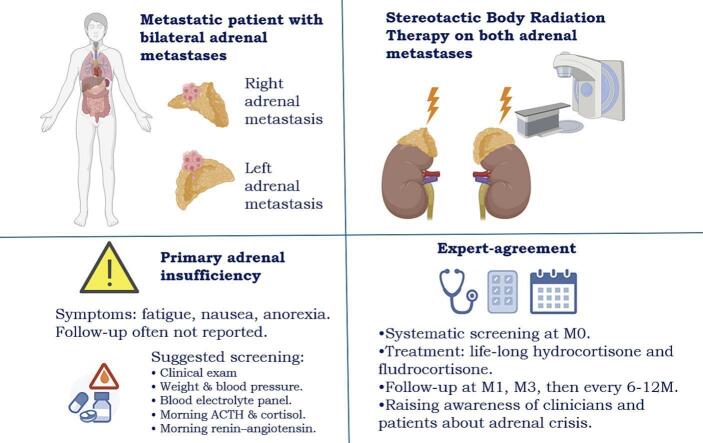

## Introduction

The adrenal gland is a common site for metastatic tumor dissemination, particularly in patients with lung and kidney cancers [1]. A 2019 review stated that adrenal gland metastases occur in up to 20% of patients with non-small cell lung cancer (NSCLC) [2]. Unilateral metastases are predominantly observed, while the occurrence of bilateral adrenal metastases ranges from 4% to 43% in surgical and stereotactic body radiation therapy (SBRT) series [3], [4]. Surgery is the main local treatment modality, but SBRT has gained popularity as an ablative non-invasive alternative treatment with a minimal occurrence of side effects, and shorter treatment time [5].

Despite excellent local control rates, adrenal insufficiency could be a serious post-treatment induced effect in case of bilateral involvement (or in case of unilateral metastasis in patients with solitary gland). There is limited data regarding its prevalence and standardized follow-up guidelines are lacking [6]. Its management after ablative therapies remains unclear; therefore, expert-based guidance may be helpful for daily practice.

The objective of this comprehensive review is to discuss the literature data on primary adrenal insufficiency (PAI) in patients with bilateral adrenal metastases receiving curative radiation therapy (RT) and define appropriate recommendations from a multidisciplinary board expert agreement.

## Material and methods

### Study design and search strategy

This work presents a comprehensive literature review on patients with bilateral adrenal gland metastases (or of unilateral metastasis in patients with solitary gland) managed by SBRT and conventional fractionated RT treated with curative intent.

This study is a comprehensive review of the literature conducted in accordance with the key principles of the Preferred Reporting Items for Systematic Reviews and Meta-Analyses (PRISMA) guidelines [7].

A literature search was performed in the PubMed and Embase databases to identify studies reporting on adrenal metastases treated with radiotherapy.

For PubMed, the search covered the period from January 1, 1956 to June 30, 2025, using a combination of Medical Subject Headings (MeSH) and free-text terms related to adrenal neoplasms and radiotherapy. The search strategy was as follows:

(“Adrenal Gland Neoplasms”[Mesh] OR “adrenal neoplasm*”[tiab] OR “adrenal tumor*”[tiab] OR “adrenal cancer*”[tiab] OR “adrenal metastasis”[tiab] OR “adrenal metastases”[tiab]) AND (“Radiotherapy”[Mesh] OR radiotherap*[tiab] OR radiation[tiab] OR “stereotactic body radiotherapy”[tiab] OR SBRT[tiab] OR SABR[tiab]).

For Embase, the search covered the period from 1977 to 2025, using the following strategy:('adrenal metastasis'/exp OR 'adrenal metastasis' OR ((adrenal/exp OR adrenal) AND (metastasis/exp OR metastasis))) AND ('radiotherapy'/exp OR radiotherapy).

The search was limited to studies involving human subjects using the Embase “human” filter.

Two independent investigators (AB and KD) performed the database searches.

### Study selection

All identified records were screened based on title and abstract. Full texts were reviewed for eligibility when appropriate.

A total of 1043 articles were identified from PubMed and 3891 articles were identified from Embase before applying inclusion and exclusion criteria.

Records were not deduplicated before screening. Therefore, all records retrieved from PubMed and Embase were screened, and duplicate citations were excluded during the screening process.

### Inclusion criteria

Studies were included if they (1) Reported on patients with adrenal metastases treated with radiotherapy (including SBRT or conventional RT), (2) Included patients with bilateral adrenal metastases, or unilateral adrenal metastasis in the context of a solitary adrenal gland, and (3) were published in English.

Case reports were eligible for inclusion due to the rarity of the condition.

Patients were grouped as a functional adrenal insufficiency–risk cohort (FAI), including those with bilateral adrenal metastases treated with bilateral irradiation (BAM-BI) and those with a solitary adrenal metastasis irradiation after prior contralateral adrenalectomy (SAMI-CA).

### Exclusion criteria

Studies were excluded if they (1) Included only unilateral adrenal metastases with both glands intact, (2) Were abstracts, conference proceedings, or posters without full data, (3) Were duplicate publications (4) palliative irradiation, and (5) Did not report relevant clinical or treatment outcomes.

### Data extraction

Data were independently extracted from eligible studies, including: study characteristics (design, year, sample size), patient population and clinical context, radiotherapy techniques, dose, and fractionation, endocrine outcomes, including primary adrenal insufficiency (PAI), diagnostic methods, management strategies, and follow-up.

Extracted data were synthesized descriptively. Data from the included studies were summarized and analyzed for descriptive statistics. Information is summarized in Table 1**.**

**TABLE 1:** Cases of bilateral adrenal metastases managed by curative ablative radiation therapy: review of literature (chronological order).

**Article**	**Study design**	**RT Dose / Fractionation**	**Age**	**Follow-up**	**Number of cases with bilateral adrenal metastases irradiated**	**Adrenal insufficiency**	**Dose/BED**	**Local control**	**Treatment**
Katoh et al. (2008) [8]	Prospective clinical study	Indication: refractory disease. 9 patients, 10 adrenal metastases. Primary: lung cancer, renal cell carcinoma.	Mean age: 67.8 years.	Median follow-up: 16 months (range, 5–21 months).	2 (1 of whom had prior contralateral adrenalectomy)	Adrenal insufficiency: not assessed. No acute nor late toxicities have been reported	Dose: 48 Gy in 8 fractions (exception of 30 Gy in 8 fractions, prior RT). Technique: HFRT, with motion management. Median BED: 76.8 Gy.(range, 41–77 Gy)	Local control: freedom-from-local-progression rate was 100% at 12 months.	Not applicable.
Chawla et al. (2009) [5]	Retrospective single-institution study	Period: 2001–2007. 30 patients with 35 adrenal lesions. Primary: lung cancer (67%), gastrointestinal cancer (13%), breast cancer (10%), others (22%). Indication: oligometastatic disease, bulky or symptomatic lesions. Lung primary in 67%. 47% male, 53% female patients.	Mean age: 62.8 years.	Follow-up: 9.8 months (range, 5–21 months).	5	Adrenal insufficiency: not assessed. No acute nor late grade 2–4 toxicities have been reported. Common mild fatigue, and grade 1 nausea was described.	Dose: 40 Gy (range, 16–50 Gy) in 4–16 fractions. Technique: SBRT. Median BED: 56 Gy (range, 22–75 Gy)	Local control: 91% at 6-month, 55% at 1-year, and 27% at 2-year.	Not applicable.
Hsieh et al. (2009) [9]	Case report	Liver cirrhosis (Child A), not a surgical candidate. Primary: HCC. Indication: Oligorecurrent metastasis.	57-year-old man.	Follow-up: >1year.	1	Adrenal insufficiency: clinically assessed. No acute nor late grade 2–4 toxicities have been reported.	Dose: Left adrenal metastasis: 54 Gy in 27 fractions followed by 48 Gy in 24 fractions. Right adrenal metastasis: 54 Gy in 27 fractions. Technique: both managed by 3D-CRT after TACE. BED: 102 Gy on the left adrenal metastasis. 64.8 Gy on the right adrenal metastasis.	Local control: partial response at 1 month follow-up on ultrasound imaging.	Not applicable.
Torok et al (2011) [10]	Retrospective single-institution case series	Period: 2002–2009. 7 patients, 9 adrenal metastases. Primary: NSCLC (57%), SCLC (14%), HCC (28%). Prior treatments: 3 patients had prior adrenal surgery. 1 patient had prior EBRT (40 Gy)	Median age: 60 years (range, 47–91)	14 months	2	Adrenal insufficiency: not assessed. No grade 3 toxicity.	Dose: Single-fraction SBRT 16 Gy (range, 10–22 Gy) Hypofractionated SBRT: 27 Gy (range, 24–36 Gy) in 3 fractions Technique: Robotic SBRT, Trilogy Linac BED: 40–60 Gy	1-year: 63%	Not applicable.
Eldaya et al. (2012) [11]	Case report	1 patient. Primary: renal cell carcinoma. Prior right adrenalectomy, before left SBRT.	55-year-old man.	Follow-up: 3 years	1 (prior contralateral adrenalectomy)	Endocrine evaluation: cortisol, ACTH. Adrenal insufficiency: none.	Dose: 40 Gy in 5 fractions Technique: SBRT with motion management. BED: 72 Gy	3-year: 100%	Not applicable.
Li et al. (2013) [12]	Retrospective single-institution cohort	Period: 2009–2012. 18 patients with adrenal metastases. Histology: adenocarcinoma and squamous cell carcinoma (26.9%), renal cell carcinoma (15.4%), urothelium carcinoma (23.1%).	Median age: 64 years. 40 year-old man (bilateral case)	Median follow-up: not reported.	At least 1 bilateral case treated (showed on their figure 3)	Adrenal insufficiency: Not assessed.	Dose: 45 Gy (range, 30–50 Gy) in 3–5 fractions. Technique: Robotic SBRT, fiducial tracking. BED: 87 Gy (range, 48–113 Gy).	Local control: 77% at 17-months follow-up.	Not applicable.
Gamsiz et al. (2015) [13]	Retrospective single-institution cohort	15 patients, 17 adrenal metastases. Only non-small cell lung cancer. Indication: Oligometastatic disease. 60% male and 40% female patients.	Median age: 56 years.	Median follow-up: 16 months.	2	Adrenal insufficiency: Assessment through clinical evaluation and serum chemistries (electrolytes). No adrenal insufficiency reported.	Dose: 30 Gy in 3 fractions. Technique: SBRT with active breathing control. BED: 60 Gy.	Local control: 86.7% at 16-months follow-up.	Not applicable.
Wardak et al. (2015) [3]	Case report	Indication: Genuine synchronous oligo-metastasis. Primary: Non-small cell lung carcinoma.	69-year-old woman.	Follow-up: ≥16 months.	1	1 G2 PAI Adrenal insufficiency: Clinical evaluation, biochemical criteria (ACTH, cortisol) Developed adrenal insufficiency (grade 2). Diagnosis: Elevated ACTH levels and low cortisol levels. Aldosterone and renin.	Dose: 40 Gy in 5 fractions, sequentially delivered. Technique: Linac-based SBRT. BED: 72 Gy.	Local control: 9 months on last-follow-up.	Glucocorticoid therapy: 20 mg daily hydrocortisone. Mineralocorticoid therapy: 0.1 mg daily fludrocortisone.
Jung et al. (2016) [14]	Retrospective multi-institutional study	Period: 2000–2012. 9 centers. 134 patients, 142 adrenal metastases. Primary: HCC. Indication: no information about oligometastatic setting. 92.5% male and 7.5% female patients.	Mean age: 59 years	Median follow-up: 10.7 months (range, 1.1–104.2 months).	8	Adrenal insufficiency: not assessed. Symptoms: Grade 2 acute toxicities (fatigue, anorexia, nausea, vomiting) in 67.2% patients and grade 3 toxicities in 3% patients. No subgroup information regarding patients with bilateral management.	Mean dose: 45 Gy (range, 20–66 Gy) and mean fraction of 2.5 Gy/f (range, 1.8–5 Gy/f). RT technique: 3D-CRT (88%), SBRT (6.3%) and IMRT (5.6%). BED: 58.5 Gy (range, 25–112.5 Gy)	Disease control rate: 93.6%.	No information.
Chance et al. (2017) [15]	Retrospective single-institutional study	Period: 2009–2015. 43 patients, 49 adrenal metastases. 77% male and 23% female patients. Primary: mostly lung carcinoma (83%)	Median age: 64 years.	Median follow-up: 16 months (range, 3–94 months).	6	Adrenal insufficiency: clinical and laboratory evaluation. 3/6 bilateral patients developed adrenal insufficiency (1 grade 1 at 6 weeks and 2 grade 2 at 4–7 months).	Dose: 60 Gy in 10 fractions (median). Technique: SABR with IGRT. BED: 96 Gy.	Local control: freedom-from local failure was 74% at 1-year.	No information.
Haidenberger et al. (2017) [16]	Retrospective single-institutional study	Period: 2006–2015. 23 patients. 65.2% and 34.8% female patients. Primary: non-small cell lung carcinoma (39.1%), renal cell carcinoma (30.4%)	Mean age: 61.5 years.	Median follow-up: 23.6 months (range 1.7–71.5 months, mean 25.0 months).	2	Adrenal insufficiency: not assessed. Nausea in 5 patients.	Dose: 20–25 Gy in 1 fraction or 36–45 Gy in 3 fractions. Technique: Cyberknife. BED: 70–95 Gy.	Local control: 95% at 1-year and 81% at 2-year.	Not applicable.
Iyengar et al. (2017) [17]	Single-institution randomized phase II trial	29 patients, 3 adrenal metastases. Primary: non–small-cell lung cancer	Median age: 63.5 years (range, 51.0–78.0)	Median follow-up: 9.6 months (range 2.4–30.2)	1	Adrenal insufficiency: not assessed. Some grade 3–4 events, not adrenal-related.	Dose: 30 Gy in 5 fractions. Technique: SABR with motion management. BED: 48 Gy	100%	Not applicable.
Toesca et al. (2018) [18]	Retrospective single-institutional cohort.	Period: 2008–2017. 35 patients with adrenal metastases. Primary: non-small cell lung cancer (48%), HCC 20%), other tumors of gastrointestinal tract (9%) Indication: Oligometastatic disease. 60% male and 40% female patients.	Mean age: 66 years.	Median follow-up: 7 months (range, 1–54 months).	4 (1 case of prior adrenalectomy)	Adrenal insufficiency: not assessed. 1 bilateral patient with adrenal insufficiency (grade 1). Patient was previously adrenalectomized. Diagnosis: Laboratorial evidence.	Dose: 40 Gy (range, 20–54 Gy) in 5 fractions (range, 1–6 fractions). Technique: SBRT with IGRT. BED: 72 Gy (range, 30–124.8 Gy)	Local control: 92.4% at 1-year and 80.8% at 2-years.	No information.
Burjakow et al. (2018) [19]	Retrospective single-institution study	Period: 2003–2015. 33 patients, 38 adrenal metastases.	Median age: 63.3 years (range, 29–86 years)	11 months	5	Adrenal insufficiency: not assessed. No grade 3 toxicity.	Dose: 48 Gy (range, 28–68 Gy) Technique: Linac-based SBRT. BED: 67.2 Gy (range, 42–108.8 Gy)	1-year LC: 56.3%, 2-year LC: 50%	Not applicable.
Figura et al. (2019) [20]	Retrospective analysis	Period: 2013–2018. 41 patients, 45 adrenal metastases. Primary: non-small cell lung cancer (51%), renal cell carcinoma (24%), small cell lung cancer (10%) Indication: Oligometastatic disease. 76% male, 24% female patients.	Mean age: 60 years.	Mean follow-up: 10.5 months.	4	Adrenal insufficiency: not assessed. No acute nor late toxicities have been reported. Common mild fatigue, and grade 1 nausea was described.	Dose: 50 Gy (range, 25–60 Gy) in 5 fractions. Technique: SBRT with SIB and adaptative positionning. BED: 100–132 Gy.	Local control: 96% at 1-year.	Not applicable.
Scouarnec et al. (2019) [21]	Retrospective single-institution cohort	Period: 2011–2018. 31 patients, 33 adrenal metastases. Primary: lung cancer (45.2%), melanoma (19.4%), renal cell carcinoma (9.7%), breast cancer (9.7%) (58% patients were strictly oligometastatic). 64.5% male and 35.5% female patients.	Mean age: 63 years.	Median follow-up: 18 months (range: 1.4–89.5 months)	2	Adrenal insufficiency: not assessed. Grade 1–2 toxicity in 42.4%: nausea, abdominal pain, vomiting, asthenia. No grade 3 toxicity reported.	Dose: 30–55 Gy in 3–9 fractions. Technique: Robotic SBRT. Median BED: 112.5 Gy (range, 45–115.5 Gy)	Local control: 96.5% at 1-year and 92.6% at 2-year.	No information.
Helis et al. (2020) [22]	Retrospective multi-institutional cohort	Period: 2005–2018: 27 patients, 29 adrenal metastases. Primary: non-small cell lung cancer (69%), HCC (24.1%) Mostly oligometastatic disease (89.7%).	Median age: 63 years.	Median follow-up: 32.9 months.	2	Adrenal insufficiency: clinically assessed. No adrenal insufficiency reported in patients with bilateral irradiation.	Median dose: 50 Gy in 10 fractions. Technique: 3D-CRT, IMRT/VMAT Median BED: 75 Gy (range, 48–102.6 Gy)	Local control: actuarial 1-year LC 86% and actuarial 2-year LC 76%.	No information.
Chen et al (2021) [23]	Retrospective single-institution study	Period: up to December 2019. 48 patients. Primary cancer: NSCLC (71.2%), colorectal cancer (7.7%).	Median age: 65.5 years (range 43–89). Mean age**:** Not reported	Median follow-up: Not explicitly reported	4	Adrenal insufficiency: not assessed.	Dose: Range: 24 Gy / 3 fx → 60 Gy / 8 fx. Technique: MR-guided adaptive SABR (SMART) BED: 43.2–105 Gy	Not reported	Not reported
Buergy et al. (2022) [24]	Multicenter retrospective cohort	196 patients, 218 adrenal metastases. Histology: adenocarcinoma (46.3%), SCLC (13.3%), squamous cell carcinoma (12.4%), melanoma (5.5%). Indication; oligometastatic disease.	Median age: not reported.	Follow-up: 48 months.	22	Adrenal insufficiency: not assessed. No ≥ grade3 toxicity reported.	Median dose: 35–50 Gy in 3–10 fractions. Technique: SBRT. BED: 65.5–96.1 Gy	Median PFS: 6.2 months vs 5.5 months (high vs low dose) (p = 0.13).	Not applicable.
McCann et al. (2022) [25]	Retrospective single-institution study	Period: 2015–2019 20 patients, 23 adrenal metastases. Primary: melanoma. Concurrent immunotherapy in 60% of patients.	Median age: 61 years.	Median follow-up: 20 months	3	Adrenal insufficiency: 2 patients with grade 2. Both had bilateral irradiation. Not explicitly graded according to CTCAE. No grade 3 toxicity reported. Adrenal insufficiency diagnosis: not reported.	Dose: 20–30 Gy in 1–12 fractions. Technique: SBAR, IMRT/VMAT, 3D-CRT Median BED10: range: 60 Gy (range, 28–94 Gy)	1-year: 64%	Steroid replacement.
Ehret et al. (2022) [26]	Retrospective bi-institutional cohort study	55 patients, 72 adrenal metastases. 14 patients (25%) were treated for more than one AGM	Median age: **66.3 years**	Median follow-up: 16.4 months Mean follow-up: 24.1 months	3 SAMI CA Bilateral irradiation not explicitly reported.	3 grade 2 adrenal insufficiency Adrenal insufficiency: hormone status before and after treatment was assessed.	Dose: Median 24 Gy (mostly single fraction) Technique: Robotic SBRT Median BED10 ≈ 80.4 Gy	92.9% at 1 year 67.8% at 2 years	All patients received hormone replacement therapy.
Elmali et al. (2022) [27]	Retrospective multi-institutional cohort.	Period: 2008–2019. 6 centers 124 patients / 146 adrenal metastases. Primary tumors: lung cancer (76%), gastrointestinal (8%), breast cancer (6%).	Median age: 60 years.	Median follow-up: 8 months (range, 1–124).	≥2 confirmed bilateral SBRT (out of 22 bilateral disease)	Not explicitly stated that all received bilateral RT. Four cases of reirradiation due to local relapse. Two patients developed adrenal insufficiency (one as grade 5, and the other not specified but reported as late toxicity). Both had bilateral irradiation. CTCAE v4.0 Diagnosis not specified.	Dose: 36 Gy (range, 15–60 Gy) in 4 fractions (range, 3–10). Technique: 3D-CRT, VMAT, IMRT, Robotic SBRT. Median BED: 65 Gy (range, 28–180).	Overall local control: 83%	Not reported.
Herndon et al. (2023) [4]	Single-center retrospective longitudinal cohort	Period: 2010–2021. 56 patients with adrenal metastases. Primary: lung cancer (73.2%), melanoma (7.1%), renal cell cancer (5.3%). 60.7% male and 39.3% female patients.	Mean age: 69.5 years.	Median follow-up: 17.9 months.	10 (9 bilateral, 1 one solitary gland)	Adrenal insufficiency: clinical assessed with endocrine evaluation. 8/10 patients developed adrenal insufficiency (6 with grade 2, 2 with grade 1). Diagnosis: Biochemical parameters and/or clinician evaluation. Time to diagnosis: 6.1 months.	Median dose: 50 Gy in 5 fractions. Technique: SBRT BED: 100 Gy	Local control: 87.5% at 19.7 months follow-up.	Glucocorticoid therapy: 15–80 mg daily hydrocortisone in 7 patients. Mineralocorticoid therapy: 0.05 mg daily fludrocortisone in 4 patients.
Schneiders et al. (2023) [28]	Single-institution retrospective cohort	Period: 2016–2023. 107 patients, 114 adrenal metastases. Oligometastatic/oligoprogressive. Lung cancer in 67.3%. 68.2% male and 31.8% female patients.	Median age: 66 years.	Median follow-up: 13.8 months.	7	Adrenal insufficiency: clinically-based assessment. 1 CTCAE v5.0 grade 2 adrenal insufficiency (bilateral irradiation).	Technique: MR-guided RT BED10 ≥ 80 Gy in 81.6% BED10 ≥ 100 Gy in 67.5%	Local control: 98.5% at 1-year and 96% at 2-years.	No information.
Haisraely et al. (2024) [29]	Single-institution retrospective study	Period: 2015–2022 83 patients. Histology: NSCLC (44%), melanoma (16.8%), colon carcinoma (12%). Indication: Oligometastatic disease in 70% patients, oligoprogression in 30% patients 54.7% male and 45.7% female patients.	Mean age: 67.1 years.	Follow-up: >2 years.	6	Adrenal insufficiency: 1 bilateral patient developed adrenal insufficiency (grade 2). No previous history of immunotherapy or previous steroid-use Symptoms: General weakness. Diagnosis: Cortisol level assessment. Time to diagnosis: 5 months.	Dose: Mean number of fractions: 5 (range, 3–10) and median dose per fraction was 8 Gy/f (range, 5–12). Technique: VMAT SBRT with IGRT, and motion management. Median BED:75 Gy (range, 48–105)	Local control: 74.6% at 2-years.	Prednisone 5 mg/day.
Hamidi et al. (2024) [30]	Single institution retrospective cohort	Period: 2007–2022. 66 patients. Primary tumors: renal cell carcinoma (41%), lung (38%), colorectal (9%). 77.8% male and 22.2% female patients.	Median age: 61 years.	Median follow-up: 17 months.	18 (9 prior contralateral adrenalectomy)	Adrenal insufficiency: 4/9 bilateral patients (2 grade 2, 1 grade 3, 1 grade 4) 4/9 in prior adrenalectomy subgroup Diagnosis: Morning Cortisol + ACTH. Time to diagnosis: 4.3 months.	Dose: 40 Gy in 5 fractions, 20 Gy in single dose, 13.5 Gyx3 fractions. Technique: SBRT (97%) with motion management. BED: 128 Gy	Local control: 82% at 6 months and 75% at 1 year.	Glucocorticoid therapy: 15–25 mg/day Bolus of 100 mg in case of adrenal crisis. Mineralocorticoid therapy in 4 patients. Fludrocortisone.
Yuste et al. (2024) [31]	Multicenter retrospective study	Period: 2010–2021. 11 centers. 110 patients, 121 adrenal metastases. Mainly non-small cell lung cancer (55.4%) Indication: Oligometastatic. 63.6% male and 36.4% female patients.	Median age: 66 years.	Median follow-up: 22.1 months.	11	Adrenal insufficiency: Not assessed. All 11 bilateral patients were documented with adrenal dysfunction during follow-up (grade unknown).	Dose: 30–45 Gy in 5 fractions. Technique: SBRT. BED: 72 Gy	Local control: 85.9% at 1-year and 72.5% at 2-years.	All bilateral adrenal patients received hydrocortisone.
Lütscher et al. (2024) [32]	Single center retrospective study.	Period: 2013–2023. 41 patients. Primary (SBRT cohort): NSCLC (39%), SCLC (4.9%), others.	Median age: 64 years.	Follow-up: 11.3 months	4 3 with bilateral RT, 1 with mixed surgery and contralateral SBRT	No adrenal insufficiency after SBRT. Diagnosis not specified (likely clinical diagnosis). AI only after bilateral adrenalectomy.	Dose: 30–40 Gy in 5 fractions Technique: Linac-based SBRT with IGRT. BED: 47.7 Gy (range, 28–65.6 Gy)	1-year LC: 70% 2-year LC: 52.5%.	Not applicable.
Urgurluer et al. (2024) [33]	Multi-institutional pooled retrospective study	11 centers Period: 2016–2022 255 patients (269 adrenal metastases). Primary: lung cancer (68.6%), genitourinary (9.8%), gastrointestinal (5.5%).	Median age: 65 years.	Follow-up: 17.7 months.	12	Adrenal insufficiency: 3 CTCAE v5.0 grade 2 1 CTCAE v5.0 grade 3. Diagnosis: not reported.	Dose: 45 Gy (range, 16–60 Gy) in 5 fractions (range, 1–8) Technique: MR-Linac BED: 100 Gy (range, 37.5–132 Gy)	1-year LPFS: 94.0% 2-year LPFS: 88.3%	Not reported.
Hoegen-Saßmannshausen et al. (2024) [34]	Retrospective single-/bi-institutional cohort study	Period: 2020 – 2024 35 patients Primary: NSCLC dominant (46%), RCC, melanoma	Median age: 67 years	Median follow-up: 18.2 months	5	Adrenal insufficiency: 2 (no grading reported) Diagnosis: not reported.	Dose: Median 50 Gy (range 30–60 Gy) MRgRT (SMART) BED: Median ∼ 100 Gy (range ∼ 60–132 Gy)	**1-year local control: 94.3%** **2-year local control: 88.2%**	Hormone replacement

### Data synthesis

Given the heterogeneity in study design, reporting, and outcome definitions, a quantitative *meta*-analysis was not performed. Instead, results were summarized using descriptive statistics.

### Risk-of-bias acknowledgment

A formal risk-of-bias assessment was not performed due to the heterogeneity of the included studies, which consisted predominantly of retrospective series and case reports, precluding the use of standardized assessment tools.

### Expert agreement process

In addition to the comprehensive literature review, we established an expert agreement process to address clinical questions insufficiently answered by existing data. This expert agreement was developed by a multidisciplinary group including radiation oncologists (n = 13), endocrinologists (n = 1), and medical oncologists (n = 2), all with experience in adrenal metastases management. One medical physicist contributed to manuscript discussions but was not involved in the expert agreement process. Based on key findings and knowledge gaps, structured clinical questions were formulated (e.g., treatment and follow-up of PAI). Experts from the AROME (Association of radiotherapy and oncology of the Mediterranean area; www.aromecancer.org) network were asked to independently provide their clinical opinion and preferred management strategies, which were then discussed collectively through group meetings and electronic correspondence. Final agreements were reached through discussion and unanimous approval. These agreements are referred to as “expert agreement” throughout the manuscript and represent practical expert opinion-based guidance in the absence of high-level evidence.

No formal Delphi process or structured evidence grading system was applied.

## Results

### Studies included

A total of 4934 articles published were identified through the search strategy and screened for eligibility. Only 30 studies were selected when inclusion and exclusion criteria were applied, including 25 retrospective studies, 1 prospective cohort, 1 phase II trial, and 3 case reports. Among studies that included bilateral or both unilateral and bilateral adrenal metastases, 1,662 patients had adrenal metastases in total, of whom 156 had bilateral adrenal metastases managed with RT or unilateral metastasis treated with RT on a solitary remaining adrenal gland. These 156 patients were included in our review. One study depicted bilateral adrenal metastases managed by SBRT in its figure legends but provided no details about other cases, therefore we included one patient with bilateral adrenal metastases managed by SBRT from this article [12].

One study had 22 bilateral adrenal metastases and explicitly confirmed irradiation of two patients with bilateral adrenal metastases; therefore, only these two cases were counted as bilateral irradiation, as information for the remaining cases was lacking [27].

We excluded studies when the bilateral irradiation was not explicitly reported.

We excluded studies when the PAI incidence was not specified if it occurred in bilateral irradiation.

We excluded patient-based analyses and focused on lesion-based reporting, including only studies that explicitly reported bilateral adrenal irradiation.

**Fig. 1:**
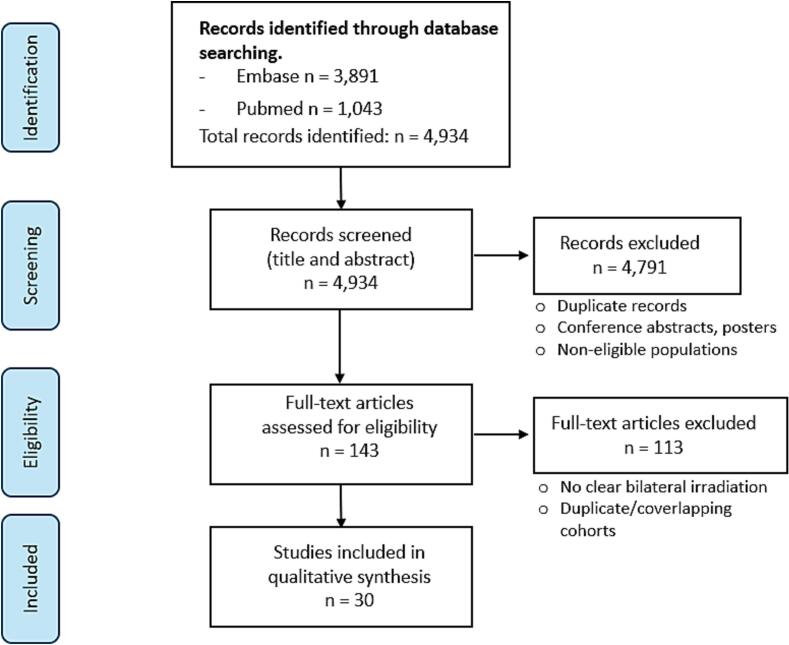
**PRISMA flow diagram of study selection.** Flowchart illustrating the process of identification, screening, eligibility, and inclusion of studies in this systematic review. A total of 4,934 records were identified through database searching (Embase, n = 3,891; PubMed, n = 1,043). After screening titles and abstracts, 4,791 records were excluded due to duplication, conference abstracts/posters, or non-eligible populations. A total of 143 full-text articles were assessed for eligibility, of which 113 were excluded mainly due to absence of clearly reported bilateral adrenal irradiation or overlapping cohorts. Finally, 30 studies were included in the qualitative synthesis.

A PRISMA diagram depicts the process of study selection in Fig. 1.

### Demographics

The specific demographics of patients with bilateral adrenal metastases were not detailed in the reviewed articles (except for case reports).

In 16 cases out of 156 (10%), patients had SAMI-CA [[8], [11], [18], [26], [30], [32]]. All other cases had BAM-BI.

Across the included studies reporting BAM-BI or SAMI-CA, median age ranged from approximately 55 to 69 years. Follow-up was generally limited, with median durations ranging from about 7 to 32 months, although a few studies reported longer follow-up exceeding 2–4 years.

The most common primary tumors were lung cancer (predominantly non–small cell lung cancer), followed by renal cell carcinoma, hepatocellular carcinoma, melanoma, and gastrointestinal malignancies.

Treatment was most frequently delivered in the setting of oligometastatic or oligoprogressive disease, with curative or ablative intent.

Mean follow-up duration, RT indications, RT doses and techniques and local control are reported in Table 1.

Across the selected studies, the use of concurrent systemic therapy, including immunotherapy, was inconsistently reported and generally limited.

The proportion of patients treated concurrently with SBRT was not consistently specified.

One study reported on steroid use [4]. In this study, 7 patients had a history of previous long-term steroid use, however there was no information on how these patients were distributed among those with bilateral adrenal metastases [4].

### Prevalence of adrenal insufficiency after bilateral adrenal radiation therapy (or at least unilateral RT on solitary gland)

A total of 156 patients from the FAI-risk cohort, including patients with BAM-BI and SAMI-CA were included for review (30 articles). Only 14 studies out of the 30 selected articles reported PAI diagnosis [[3], [4], [9], [11], [13], [15], [18], [22], [26], [28], [29], [30], [31], [32]]. PAI diagnosis not reported in 4 studies [[25], [27], [33], [34]].

Out of these 156 patients, forty-seven (30%) were reported with diagnosed PAI after irradiation. In 109 patients (70%), no adrenal-related toxicities were reported or information was missing.

Among the 47 patients diagnosed with PAI, 5 had grade 1 [[4], [15], [18], [30]], 23 had grade 2 [[3], [4], [15], [25], [26], [28], [29], [30], [33]], 2 had grade 3 [[30], [33]], 2 had grade 4 [30], and 1 had grade 5 PAI [27]; the grade was unknown in 14 patients [[27], [31], [34]].

Four studies assessed adrenal insufficiency in their follow-up and did not report PAI [[11], [13], [22], [32]].

When documented, PAI generally occurred within the first months following adrenal irradiation.

We excluded studies with bilateral adrenal metastases when bilateral irradiation was not explicitly stated, as it was not possible to determine whether both adrenal lesions were irradiated, or whether one lesion corresponded to recurrence or remained untreated.

### Diagnosis of primary adrenal insufficiency

Forty-seven patients from the FAI-risk cohort (BAM-BI and SAMI-CA) were diagnosed with PAI following adrenal irradiation.

When reported, diagnosis was based on clinical assessment [[3], [4], [9], [13], [15], [22], [28], [29], [32]], on biochemical parameters/serum electrolytes [[3], [4], [13], [15]], or on endocrine evaluation [[3], [4], [11], [26], [29], [30]]. Diagnosis was not clearly specified in one study [31].

Only 14 out of 30 of the selected studies explicitly reported primary adrenal insufficiency assessment or diagnosis, with heterogeneous methods including clinical evaluation, biochemical testing, and CTCAE-based reporting, while the majority did not report systematic endocrine assessment. Some studies did not assess adrenal insufficiency, but reported adrenal insufficiency during their follow-up.

Therefore, we acknowledge that the reported 30% PAI incidence represents a descriptive proportion of reported cases rather than a true pooled incidence, and may underestimate the actual risk.

### Treatment of adrenal insufficiency

Seven studies reported management of PAI [[3], [4], [25], [26], [29], [30], [31]]. Only 33 patients had reported PAI treatment.

Among the 47 patients diagnosed with PAI in the FAI-risk cohort (BAM-BI and SAMI-CA), treatment consisted of first-line glucocorticoid replacement therapy in 28 patients [[3], [4], [29], [30], [31]], followed by mineralocorticoid replacement therapy in a subgroup of 9 patients [[3], [4], [30]]. General steroid replacement alone was reported in 5 patients [[25], [26]]. There was no information on PAI management in 14 patients.

Glucocorticoid replacement therapy consisted of hydrocortisone 15–80 mg/day (16 patients) [[3], [4], [30]], or prednisone 5 mg/day (1 patient) [29]. Hydrocortisone dose was not specified in 11 patients [31]. Mineralocorticoid replacement therapy consisted of fludrocortisone 0.1 mg/day in 1 patient [3], 0.05 mg/day in 4 patients [4], and dose was lacking in 4 patients [30].

Steroid replacement therapy was not specified in 5 patients [[25], [26], [34]].

Only a limited number of studies reported management of primary adrenal insufficiency, most commonly consisting of glucocorticoid replacement (e.g., hydrocortisone or prednisone), with mineralocorticoid supplementation (fludrocortisone) described in selected cases; however, treatment details were inconsistently reported across studies.

### Follow-up

Only two out of the 30 selected studies proposed a structured follow-up strategy, underlining the lack of consensus in post-RT monitoring [[4], [30]].

## Discussion

### Efficacy

Adrenal metastases may arise from different types of cancer (mainly from lung, renal and pancreatic cancer). In 1990, it was hypothesized that in the early metastatic setting of lung cancer, adrenal metastases may result from direct lymphatic spread via retroperitoneal channels, or that they may later develop by hematogenous spread [[35], [36]]. Most patients develop adrenal metastases when they already have hematogenous spread to other organs [37]. Several ablative treatment modalities are available: surgery (open or laparoscopic adrenalectomy), radiofrequency ablation, cryotherapy, transcatheter arterial chemoembolization, and SBRT [[37], [38]].

Surgery remains the standard of care for adrenal metastases, but SBRT is increasingly used as an ablative technique when surgery is not indicated [3]. Historically, RT was used in palliative settings, to treat painful or hemorrhagic adrenal metastases [37]. The recent concept of oligometastatic disease paired with the evolution of diagnostic imaging methods and modern RT techniques such as intensity-modulated radiation therapy (IMRT), real-time image-guided radiation therapy (IGRT) volume-modulated arc therapy (VMAT) and SBRT played a significant role contributing to a paradigm shift in metastatic disease [[37], [39], [40]]. SBRT can be delivered with curative intent for local control and currently stands as an alternative ablative therapy alongside surgery for adrenal metastases.

The optimal SBRT dose and fractionation schedules in oligometastatic disease are still under investigation [41]. In a recent report by the OligoCare prospective EORTC registry of oligometastatic disease, adrenal gland metastases were treated with notably lower doses than those used for other lesions [41]. Estimated cancer-specific BED for adrenal metastases (using α/β = 10 Gy) was 60 Gy (99.8% CI, 13–106) [41]. Higher BED appears however to be associated with higher control rates. Chen et al. reported local control rates of 92.9% at 1-year follow-up with a BED of 100 Gy, 84.8% with a BED of 80 Gy, and 70.5% with a BED of 60 Gy (α/β = 10) [42]. SBRT doses for adrenal metastases are lower compared to other metastatic sites due to their proximity with critical radiosensitive organs at risk, such as the duodenum, bowel, spinal cord, and kidney [43].

Of the 156 patients of our FAI cohort, all received treatment to adrenal metastases with curative intent. The SABR-COMET study reported in 2019 that SBRT was associated with an improvement in overall survival when all oligometastatic lesions were irradiated (hazard ratio 0·57, 95% CI 0·30-1·10; p = 0·090) [44]. A total of nine adrenal metastases were included, two in the control group and seven treated with SABR, highlighting the relative underrepresentation of adrenal lesions in prospective randomized data [44]. No specific information was given if patients had undergone bilateral adrenal irradiation, therefore this study was not included in our review in Table 2 [44].

**Table 2:** Expert agreements for bilateral adrenal insufficiency management.

**CTCAE v5.0 Grade**	**Symptoms**	**Recommended treatment**	**Recommended follow-up**	**Supporting level of evidence/Strength of recommendations based on author consensus**
1	Asymptomatic; clinical or diagnostic observations only.	In case of glucocorticoid deficiency: Life-long glucocorticoid replacement therapy with 15–25 mg/day hydrocortisone that can be divided into two or three doses. In case of mineralocorticoid deficiency: Life-long mineralocorticoid replacement therapy with 0.05–0.1 mg/day of fludrocortisone.	Regular follow-up at 1 month, 3 months, at 6 months, then every 6–12 months: clinical examination, weight, blood pressure, blood electrolyte panel, assessment for signs of hypercorticism, morning plasma renin level*.	Low/Strong [[30], [45]]
2	Moderate symptoms: medical intervention indicated.	In case of glucocorticoid deficiency: Life-long glucocorticoid replacement therapy with 15–25 mg/day hydrocortisone that can be divided into two or three doses. In case of mineralocorticoid deficiency: Life-long mineralocorticoid replacement therapy with 0.05–0.1 mg/day of fludrocortisone.	Regular follow-up at 1 month, 3 months, at 6 months, then every 6–12 months: clinical examination, weight, blood pressure, blood electrolyte panel, assessment for signs of hypercorticism, morning plasma renin level*.	Low/Strong [[30], [45]]
3	Severe symptoms: hospitalization indicated.	Administration of an initial bolus of 100 mg IV hydrocortisone, followed by 50–100 mg every 6–8 h, along with isotonic saline (NaCl 0.9%) infusion. After stabilization, transition to long-term glucocorticoid replacement therapy with 15–25 mg of hydrocortisone daily, that can be divided into two or three doses. In case of mineralocorticoid deficiency: Life-long mineralocorticoid replacement therapy with 0.05–0.1 mg/day of fludrocortisone.	Regular follow-up at 1 month, 3 months, at 6 months, then every 6–12 months: clinical examination, weight, blood pressure, blood electrolyte panel, assessment for signs of hypercorticism, morning plasma renin level*.	Low/Strong [45]
4	Life-threatening consequences: urgent intervention indicated.	Administration of initial bolus of 100 mg IV hydrocortisone, followed by 50–100 mg every 6–8 h, along with isotonic saline (NaCl 0.9%) infusion. After stabilization, transition to long-term glucocorticoid replacement therapy with 15–25 mg of hydrocortisone daily, that can be divided into two or three doses. In case of mineralocorticoid deficiency: Life-long mineralocorticoid replacement therapy with 0.05–0.1 mg/day of fludrocortisone.	Regular follow-up at 1 month, 3 months, at 6 months, then every 6–12 months: clinical examination, weight, blood pressure, blood electrolyte panel, assessment for signs of hypercorticism, morning plasma renin level*.	Low/Strong [45]
5	Death.	Not applicable.	Not applicable.	Not applicable.

*: only in case of diagnosed mineralocorticoid deficiency.

**Fig. 2:**
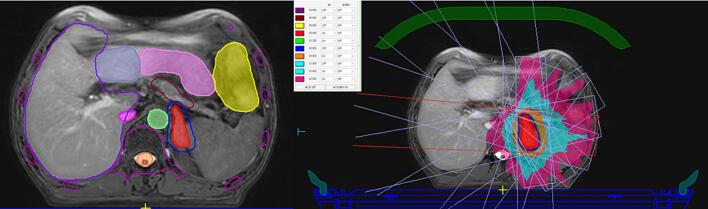
**Treatment planning for stereotactic body radiation therapy (SBRT) of an adrenal metastasis on Magnetic Resonance Imaging (MRI)-Linac. (Left)** MRI-based target and organ-at-risk delineation: gross tumor volume (GTV, red), liver (purple), stomach (yellow), kidney (green), duodenum (pink), and spinal cord (orange). **(Right)** Corresponding SBRT treatment plan with dose distribution. Isodose lines illustrate conformal coverage of the adrenal target while sparing surrounding critical organs. 95% isodose level in green colorwash. A SBRT treatment plan using eleven coplanar fields was generated, with dose calculation performed using a full Monte Carlo algorithm (Unity, Elekta, Sweden). A fully online adaptive MR-guided radiotherapy (MRgRT) workflow was selected due to the anatomical location of the target lesion. Intra-fraction motion was controlled by an automatic gating system triggered by target displacement and monitored with real-time cine-MRI. The prescribed dose was 35 Gy in five fractions of 7 Gy to the PTV, in accordance with ICRU 91 guidelines. All scheduled fractions required re-optimization because of significant variations in dose distribution affecting both organ-at-risk constraints and target coverage. (For interpretation of the references to colour in this figure legend, the reader is referred to the web version of this article.)

Magnetic resonance–guided linear accelerators (MRI–Linacs), particularly in the form of stereotactic magnetic resonance–guided online adaptive radiotherapy (SMART or MRgSBRT), have emerged as a highly precise and adaptive approach for treating adrenal metastases [46]. They offer superior soft–tissue contrast, real–time intrafraction tumor tracking, and daily plan adaptation, enabling safe dose escalation even for lesions bordering sensitive gastrointestinal organs such as the stomach or duodenum (Fig. 2) [46].

### Toxicity

Despite the growing use of RT in patients with BAM-BI or SAMI-CA, data on its impact on adrenal function remain scarce.

Nevertheless, SBRT has proven to be an effective and well-tolerated treatment for oligometastatic patients, with overall low toxicity rates [41]. However, data on PAI in patients with BAM-BI or SAMI-CA are lacking.

To the best of our knowledge, this is the first review of PAI assessment following bilateral irradiation for adrenal metastases. PAI should not be underestimated, especially given the increasing number of long-term survivors and the potential life-threatening risk related to acute PAI. In these reports, the true rate of PAI could be underestimated, as some patients may have reported symptoms compatible with PAI (e.g. fatigue, anorexia, nausea, vomiting) without specific hormonal dosage. Moreover, very few of our included studies provided relevant data on the management of post-treatment PAI [[4], [30]].

Data on adrenal toxicity outcomes are limited following SBRT [3]. This is in accordance with our findings in this report as most of our selected studies did not assess adrenal insufficiency **(**Table 1**)** [[5], [8], [10], [12], [14], [16], [17], [18], [19], [20], [21], [23], [24], [31]]. In some cases, PAI diagnosis was reported [[25], [27], [33], [34]].

PAI is likely to occur when 90% of both adrenal glands become non-functional [[3], [9]]. The timeline for developing PAI after SBRT, along with the threshold dose and dose–effect relationship, remain unclear [3]. In case of unilateral adrenalectomy, up to 22% may experience post-operative PAI highlighting the absence of a consistent dose/response pattern [3].

It should be noted that adrenal insufficiency in this setting is likely multifactorial and may result not only from radiotherapy, but also from tumor-related adrenal destruction, prior adrenal surgery, systemic steroid exposure, and concurrent systemic therapies.

In the current report, among 156 patients with bilateral adrenal metastases (or at least with solitary gland managed by RT) managed by SBRT, 47 patients (30%) were reported with PAI.

Bilateral adrenal irradiation and irradiation of a solitary remaining adrenal gland represent distinct clinical scenarios that may carry different risks of primary adrenal insufficiency, and ideally should be analyzed separately.

However, due to the limited and heterogeneous nature of the available data, as well as the lack of consistent reporting at the individual patient level, separate analyses were not feasible.

We therefore chose to group these populations based on a shared underlying concept, namely the absence of sufficient functional adrenal reserve, which places patients at risk of primary adrenal insufficiency.

Accordingly, patients were grouped into a FAI-risk cohort, including those with BAM-BI and those with a SAMI-CA.

The primary tumor distribution among patients who developed PAI was not reported separately in the included studies, and individual patient-level data were generally not available. As a result, it was not possible to perform a comparative analysis between patients who developed PAI and the overall population treated with bilateral adrenal irradiation.

One study reported PAI in 50% patients treated to both adrenal glands with a median follow-up of 16 months [15]. These results should be taken with caution due to the very small number of patients irradiated for both adrenal metastases (6 patients). Given that adrenal glands are at high risk of becoming dysfunctional after SBRT [3], [4], it is unlikely that post-treatment PAI in FAI group occurs in only 30% cases (according to our current review) or 50% cases [15].

Among the scarce reporting of post-treatment PAI diagnosis, there is also a lack of information on its management. Thirty-three patients out of 47 patients with post-SBRT PAI (70%) had reported treatment [[3], [4], [25], [26], [29], [30], [31], [34]]. Data was lacking in 30%.

The underreporting of PAI may also be explained by the use of steroid-based premedication in chemotherapy protocols, which can mask its clinical manifestations. Since SBRT is increasingly used in the oligometastatic setting, the potential impact of bilateral adrenal irradiation on adrenal function may be underestimated, particularly in patients receiving systemic treatments.

After systematically reviewing all included studies (n = 30), we found that no study has robustly identified or validated dosimetric predictors of primary adrenal insufficiency (PAI).

In the most informative endocrine-focused cohorts, PAI was strongly associated with bilateral adrenal irradiation or prior contralateral adrenalectomy, rather than radiobiological parameters [[4], [30]].

Other studies that reported PAI did not perform formal dose–toxicity adrenal-related analyses [[15], [25]].

Importantly, the vast majority of included studies (including large multicenter series such as Buergy et al.) did not assess adrenal function at all, precluding any meaningful dosimetric analysis of endocrine toxicity [24]. Even in cohorts with a substantial number of bilateral cases, adrenal insufficiency was frequently unreported, likely reflecting underdiagnosis rather than absence of toxicity.

Taken together, the available evidence suggests that (1) PAI is primarily driven by loss of functional adrenal reserve, particularly in the setting of bilateral irradiation or solitary adrenal glands. (2) No dosimetric parameter has been identified to predict or mitigate PAI risk.

To date, no evidence demonstrates that reducing radiation dose decreases the incidence of adrenal insufficiency. Multiple studies consistently show a dose–response relationship for local control, with inferior outcomes observed at lower BED levels.

Therefore, based on current evidence, dose reduction cannot be recommended as a strategy to mitigate PAI.

Only two articles suggested a follow-up protocol for future cases [[4], [30]]. More systematic and prolonged follow-up may increase the detection of PAI in the BAM-BI and SAMI-CA settings.

**Fig. 3:**
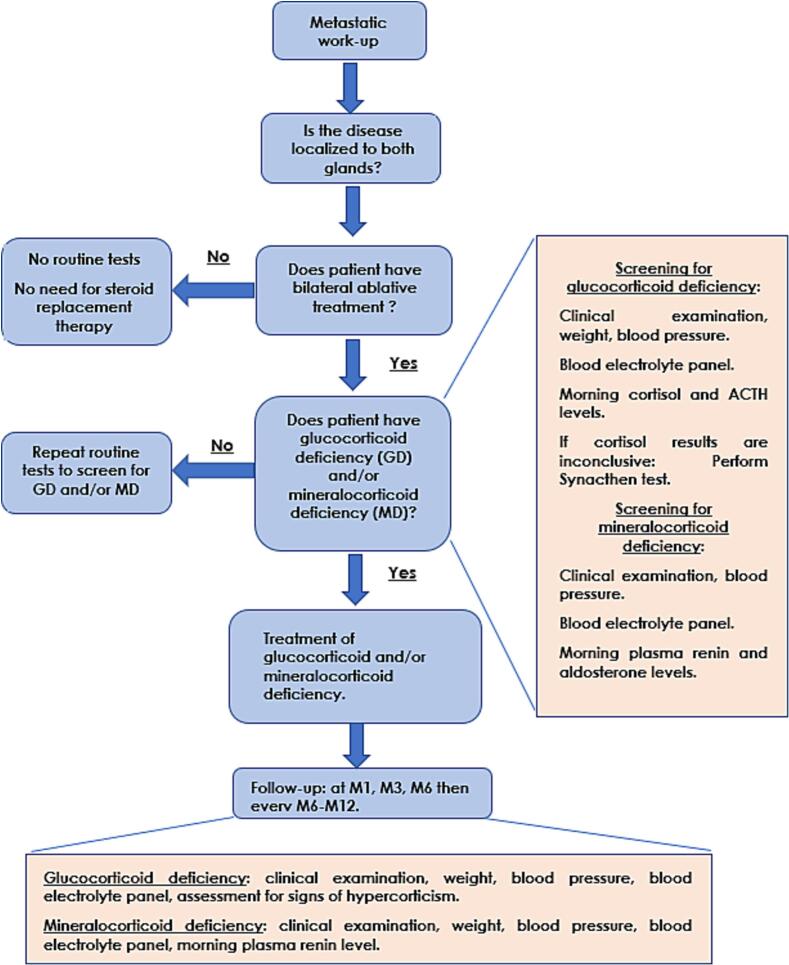
**Algorithm for adrenal insufficiency screening and management following bilateral adrenal radiation therapy.** Abbreviations: SBRT = stereotactic body radiation therapy; GD = glucocorticoid deficiency; MD = mineralocorticoid deficiency; ACTH = adrenocorticotropic hormone; M = month (follow-up timepoint). The flowchart outlines the stepwise approach to identifying and managing glucocorticoid and/or mineralocorticoid deficiency in patients with bilateral adrenal disease undergoing ablative radiotherapy. Screening is recommended for patients treated on bilateral adrenal metastases (or at least having unilateral on a solitary gland), using clinical examination, electrolyte panels, and hormonal assays (morning cortisol, ACTH, renin, aldosterone). In case of adrenal insufficiency (glucocorticoid and/or mineralocorticoid deficiency), we recommend long-life steroid replacement therapy. Follow-up should be at regular intervals (M1, M3, M6, then every 6–12 months). This includes clinical examination, weight, blood pressure, blood electrolyte panel, assessment for signs of hypercorticism, morning plasma renin level (in case of mineralocorticoid deficiency).

Overall, this review highlights the lack of assessment, reporting and data of post-treatment PAI management, occurring in the BAM-Bi and SAMI-CA settings. In Fig. 3, we propose a medical decision-making flowchart for the evaluation and management of PAI in the BAM-Bi and SAMI-CA settings.

## Limitations

Our review has several limitations. First, the available data is scarce and largely retrospective, with case reports and heterogeneous study designs that limit direct comparisons. We included 25 retrospective studies, 1 prospective cohort, 1 phase II trial, and 3 case reports, highlighting the scarcity of available data. Publication bias is also likely, as we excluded 959 nine unpublished datasets (934 abstracts and 25 conference paper) with adrenal metastases treated with radiotherapy. This suggests that the lack of reporting may be even greater than what we observed.

Second, the heterogeneity of study populations, treatment protocols, and reported outcomes further complicates consistent interpretation. Most studies were not specifically designed to evaluate adrenal function, and only rarely did they provide details on bilateral adrenal irradiation.

Finally, the incidence of PAI reported in this review must be interpreted cautiously. The available literature is limited by substantial heterogeneity in study design, follow-up, endocrine assessment, and toxicity reporting. In many studies, adrenal insufficiency was not specifically assessed, while in others the diagnostic work-up was based on non-uniform clinical and/or biochemical criteria. As a result, underdetection and reporting bias are likely. Therefore, the reported incidence should be viewed as a descriptive signal from the existing literature rather than a precise pooled estimate of risk.

We did not consider a formal pooled incidence estimate to be methodologically robust because PAI ascertainment was inconsistent, often absent, and definitions varied across studies. We therefore report the incidence descriptively and emphasize the uncertainty around the true rate.

### Expert agreements

To address the lack of standardized guidelines for managing PAI after bilateral adrenal SBRT, we developed expert-based recommendations. A multidisciplinary panel of radiation oncologists, endocrinologists, and medical oncologists reviewed the available evidence and shared their clinical experience.

These proposals were subsequently reviewed and refined through group discussions and electronic exchanges. Final recommendations were established by definitive agreement among all participants, reflecting current clinical practice in the absence of high-level evidence.

The resulting agreements, although not derived from a formal Delphi process, were formally approved by all experts. It provides practical, real-world guidance to support clinical care in the absence of high-level evidence [[45], [47]] **(**Table 2**).**

### Expert agreements for adrenal insufficiency assessment

In our review, when assessment was reported by studies, the specific work-up was not specified. There is little available data regarding post-radiation follow-up measures [4]. Baseline endocrinology evaluation with follow-up should be therefore necessary [3]. Among patients with bilateral ablative adrenal metastases management, 28% may experience an adrenal crisis during their lifetime [3].

• After unilateral ablative adrenal metastases with an intact and untreated contralateral adrenal gland, we do not recommend routine endocrine tests nor steroid replacement therapy [[3], [47]].
• After ablative treatment for bilateral adrenal involvement:
- we recommend screening glucocorticoid deficiency with a clinical examination, and by measuring blood, weight, blood electrolyte panel, morning cortisol and morning ACTH. Blood tests should be performed before treatment. In case of no glucocorticoid deficiency, repeat routine tests at 1 month, 3 months, 6 months, and then every 6–12 months. The Synacthen (ACTH stimulation test) test should be used as a second-line test in case of inconclusive morning cortisol test [47].
- we recommend screening for mineralocorticoid deficiency with a clinical examination, and by measuring blood, weight, blood electrolyte panel, morning renin/aldosterone plasma activity [47].

Fig. 3 presents the literature-based algorithm for PAI screening and management following bilateral adrenal RT.

*Recommendations for adrenal insufficiency treatment* **(**Table 2**)**

• In case of symptomatic glucocorticoid deficiency (biological and/or clinical), life-long glucocorticoid replacement therapy with 15–25 mg of daily hydrocortisone (depending on height and weight and on the severity of cortisol deficiency) should be given (10 to 15 mg in the morning and 5 to 10 mg at lunch) [[30], [45]].
• In case of mineralocorticoid deficiency, we recommend life-long mineralocorticoid replacement therapy with 0.05–0.1 mg of daily fludrocortisone) [45].
• In case of adrenal crisis: we recommend administration of an initial bolus of 100 mg IV hydrocortisone, followed by 50–100 mg every 6–8 h, along with isotonic saline (NaCl 0.9%) infusion. After stabilization, transition to long-term glucocorticoid replacement therapy with 15–25 mg of daily hydrocortisone, which can be divided into two or three doses) [45].

### Recommendations for adrenal insufficiency follow-up

After PAI following post-bilateral SBRT (or at least unilateral SBRT on a solitary gland), we recommend regular follow-up at 1 month, 3 months, at 6 months, then every 6–12 months. This includes clinical examination, weight, blood pressure, blood electrolyte panel, assessment for signs of hypercortisolism, morning plasma renin level (in case of mineralocorticoid deficiency) [45] **(**Table 2**,** Fig. 3**).**

### Medical education

Medical education on adrenal crises in PAI is crucial for both healthcare practitioners and patients particularly regarding early signs such as asthenia, gastrointestinal symptoms, hyponatremia, and hyperkalemia [47].

Healthcare professionals must be trained to recognize the early signs and symptoms of an adrenal crisis, as timely intervention is vital to prevent life-threatening complications [47]. Education should also extend to patients and their care providers, ensuring they understand the importance of adherence to steroid therapy, how to adjust their medication during illness or stress, and when to seek emergency medical help. Comprehensive education programs can significantly reduce the incidence of adrenal crises and improve patient outcomes.

## Conclusion

PAI following bilateral adrenal SBRT remains under-recognized and under-reported. In our review, we found an incidence of 30%, highlighting that this complication is not rare. However, interpretation is limited by heterogeneity of studies and the lack of systematic adrenal function assessment.

Despite the limited available data, we propose practical recommendations based on a multidisciplinary expert agreement, including a pragmatic workflow for screening and managing primary adrenal insufficiency in patients treated with bilateral adrenal SBRT, to guide clinical practice. These expert-based recommendations, developed through a multidisciplinary agreement, aim to provide real-world guidance in the absence of standardized guidelines. Ultimately, prospective studies are needed to better define the true incidence and optimize management, but until then, systematic monitoring is essential to improve patient safety and care.

The main contribution of this work lies in raising awareness of this at-risk population, highlighting the lack of systematic endocrine assessment, and proposing a structured screening and follow-up strategy adapted to this context.

## Funding

This research did not receive any specific grant from funding agencies in the public, commercial, or non-profit sectors.

## Declaration of Competing Interest

The authors declare that they have no known competing financial interests or personal relationships that could have appeared to influence the work reported in this paper.
